# Contamination Associated With Glove Changing Techniques in the Operating Theatre

**DOI:** 10.3389/fsurg.2022.839040

**Published:** 2022-03-22

**Authors:** Pamela Boekel, Eugene T. Ek

**Affiliations:** ^1^Department of Orthopaedic Surgery, Austin Hospital, University of Melbourne, Melbourne, VIC, Australia; ^2^Melbourne Orthopaedic Group, Melbourne, VIC, Australia; ^3^Department of Surgery, Monash Medical Centre, Monash University, Melbourne, VIC, Australia

**Keywords:** infection prevention, surgical site infection, healthcare-associated infection, surgery, sterile field, personal protective equipment, Orthopaedic Surgery

## Abstract

**Background:**

Sterility of the operative field during surgery is imperative in reducing the risk of infection. Most commonly, double gloves are worn by surgeons. When contamination occurs, the top gloves are changed intra-operatively. No studies have investigated which glove changing technique is best. Therefore, in this study, we aim to identify which top glove changing technique causes the least surface contamination.

**Methods:**

Glitterbug™ (UV fluorescent powder) was applied to the top gloves of 3 individuals who changed their top gloves according to a randomised method – Method 1: 3 pairs worn, remove the outer pair; Method 2: 2 pairs worn, remove the top glove, replace unassisted; and Method 3: 2 pairs worn, remove the top glove, and replace assisted by a scrub nurse. A blinded investigator inspected for Glitterbug™ contamination under UV light.

**Results:**

Two hundred and ten trials were performed and two types of contamination were identified, namely, direct contact and airborne spread. For absolute contamination, Method 1 had 59/64 (92%) contaminated trials, Method 2 had 49/65 (75%) contaminated trials, and Method 3 had 64/81 (79%) contaminated trials. This was statistically significant (*p* = 0.031). For direct contamination only, Method 1 had 38/64 (59%) contaminated trials, Method 2 had 24/65 (37%) contaminated trials, and Method 3 had 20/81 (25%) contaminated trials. This was statistically significant (*p* < 0.0001).

**Conclusions:**

Method 2 had a statistically significant lower contamination rate overall, with Method 3 having the lowest direct contamination rate. We believe that wearing 2 gloves, removing the top glove and replacing it, either assisted or unassisted, could decrease surface contamination of the sterile field.

## Introduction

Deep prosthetic joint infection in Orthopaedic Surgery is a devastating complication. It can often require multiple revision surgeries, prolong antibiotic therapy, and cause considerable patient morbidity and cost to the healthcare system (1, 2). Maintaining the sterility of the operative field is imperative to reduce infection risk as it has been shown that most acute prosthetic joint infections have their genesis at the time of surgery (3). One method shown to reduce surgical site infection is the practise of “double gloving” (4), where 2 pairs of gloves are worn to act as an added sterile barrier between the surgical site and the surgeon. Top gloves are routinely changed after known contamination (e.g., after inadvertently touching a non-sterile surface with a glove), after presumed contamination (such as after draping), and prior to handling prostheses (5). Anecdotally, there are many methods for changing top gloves, but there is no published evidence investigating which method creates the least amount of outer surface cross contamination.

Three commonly used methods of glove changing were identified. Method 1 involves wearing 3 pairs of gloves and removing the outer pair after potential contamination (e.g., prepping and draping). Method 2 is where 2 pairs of gloves are worn, and the top pair is removed after contamination and replaced by the individual wearing them. Method 3 is where 3 pairs of gloves are worn, and the top pair is removed and replaced with the assistance of a scrub nurse.

Each of these methods have their advantages and disadvantages. Method 1 could cause potential contamination by using a clean under glove to remove the contralateral hand's outer layer, thereby potentially touching a contaminated surface. This can happen with the other methods when doffing the top pair of gloves. However, any contamination would then be covered by a new outer glove with Methods 2 and 3.

Method 3 relies on the assumption that the scrub nurse has no outer surface contamination. Furthermore, the force required for assisted glove donning with Method 3 can potentially create an aberrant air flow. Particularly, the hand creates turbulent air flow from the glove up the forearm, potentially carrying with it any cells from the fingertips and causing airborne contamination.

However, as previously mentioned, there is still no clear evidence as to which of these techniques is most effective. We hypothesised that Method 2, whilst having the potential of both direct and airborne contamination, probably had the least risk of outer surface contamination when compared to Methods 1 and 3 as the outer surfaces were covered with a new sterile glove and less force and, therefore, less turbulent air flow is required to self-don a glove.

This study aims to identify which top glove changing technique results in the least contamination in a simulated surgical environment.

## Methods

Contamination was simulated using Glitterbug™ powder (Arrow Scientific Pty Ltd, Lane Cove, NSW, Australia), a UV fluorescent powder which is of similar particulate size and density as *Staphylococcus* aureus and skin squamous cells, the most common contaminants (4, 6). This has been validated for use in this manner by multiple other similar published studies assessing contamination by skin flora (7–11).

Three surgical trainees with extensive experience with donning of surgical gloves were recruited for this trial. Each of the trainees routinely used a different method of glove application to one another in their everyday practise. This was deliberately chosen in order to minimise performance bias. The participants applied their gloves according to a pre-determined randomised method. An online random integer generator (12) was used to create the randomisation.

**Figure 1:**
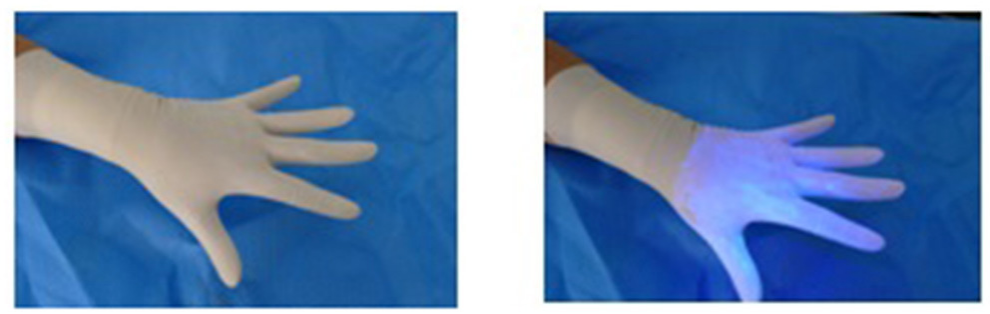
Glitterbug™ coating the top glove.

Glitterbug™ powder was then coated over the participant's outer gloves (Figure 1). Particularly, on the palmar and dorsal aspect and to the level of the palmar crease. The participant was then inspected using an UV A lamp and any contaminants present prior to the trial were removed. The participant then changed their gloves according to the pre-determined randomised method (Table 1).

**Table 1:** This table describes the 3 different methods of glove application investigated in this study.

**Glove changing methods**	
Method 1	The “three pair method”: wear 3 pairs of gloves and remove the top pair following contamination.
Method 2	The “unassisted method”: wear 2 pairs of gloves, surgeon removes the top pair of gloves following contamination, then dons a new pair, unassisted.
Method 3	The “nurse assisted method”: wear 2 pairs of gloves, surgeon removes the top pair of gloves following contamination, then dons a new pair with the assistance of a scrub nurse. The nurse holds the glove open via the cuffs, for the surgeon to place their hand inside without touching the outer surface of the glove.

**Figure 2:**
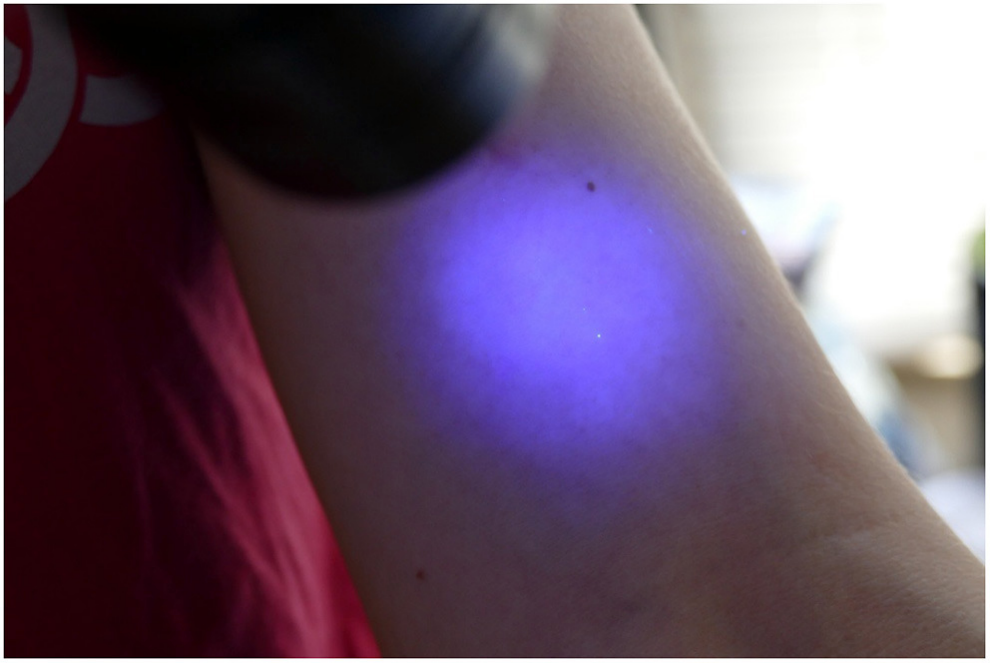
Small spot (1 × 1mm) of contamination.

**Figure 3:**
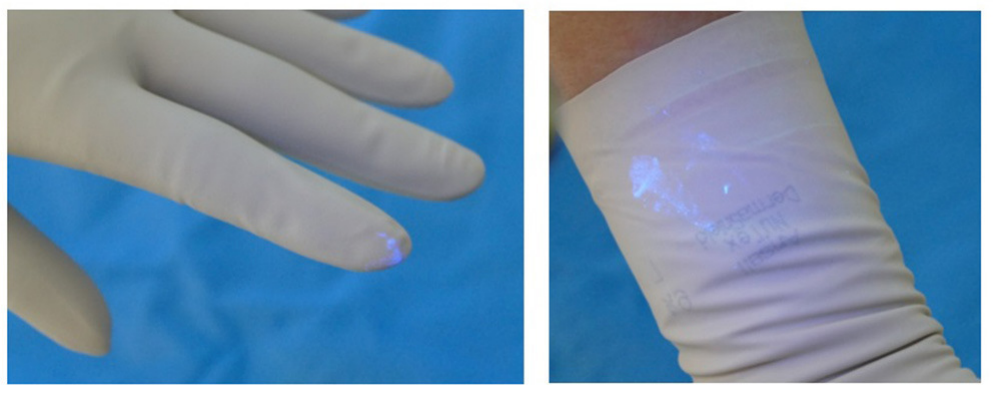
Large “smear” of outer surface contamination.

The participant then presented to the blinded investigator for inspection with another UV A lamp. The investigator noted the presence of any contaminants (present or not), the location of the contaminants (dorsum or palmar aspect of hand, the location on the hand or fingers and which finger; distance in centimetres up the arm from the level of the palmar crease), and the size and nature of the contamination, e.g., dots or speckles from airborne spread of Glitterbug™ as demonstrated in Figure 2, or a smear from direct contact of a contaminated surface on the sterile one (Figure 3), and the dimensions of the contaminant in millimetres (if greater than 1 × 1 mm). Participants' eyes were closed during the inspection to prevent performance bias.

“Absolute contamination” was used to describe any trial which had any Glitterbug™ present on outer surfaces due to either direct contact or airborne spread. “Relative contamination” was used to describe trials where the contamination was only from direct contact of contaminated surfaces. The researchers believed that distinguishing the two patterns of contamination was important as movements which cause more direct contamination can easily be changed compared to control over airborne particles.

As there were no previous studies investigating this question, a pilot study of 60 trials were performed. *Post-hoc* sample size calculations were then applied to the results from the pilot study. Thus, an estimate of the required number of trials to reach statistical significance was obtained. The *post-hoc* sample size calculation from the pilot study was 210 trials. Hence, an additional 210 trials were performed in 2 separate sessions with the same participants. This allowed the investigators to determine the required number of gloves and other consumables needed to undertake the study.

Ethical approval was sought and granted for this project via the local hospital Ethics Committee. The data was analysed using the SPSS (13) statistical analysis program. A one-way analysis of variance (ANOVA) test was used to compare the 3 trial groups. This was also used to compare the participants' results to one another. A *p* value of < 0.05 was considered statistically significant.

## Results

**Figure 4:**
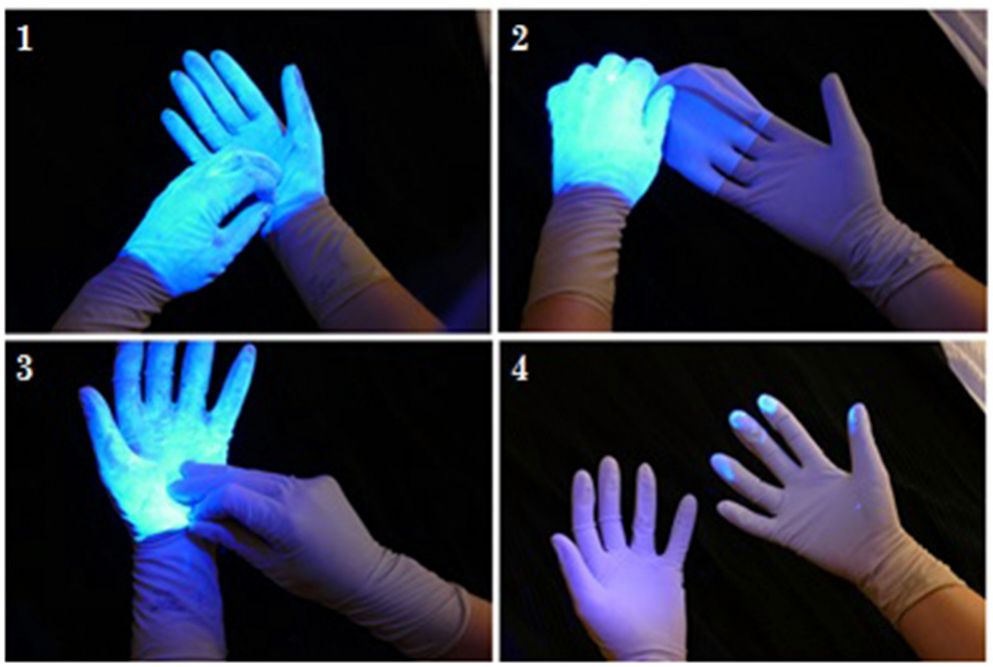
An example of how direct contamination can occur. The clean outer surface of a fresh top glove can touch the outer surface of a contaminated glove.

**Figure 5:**
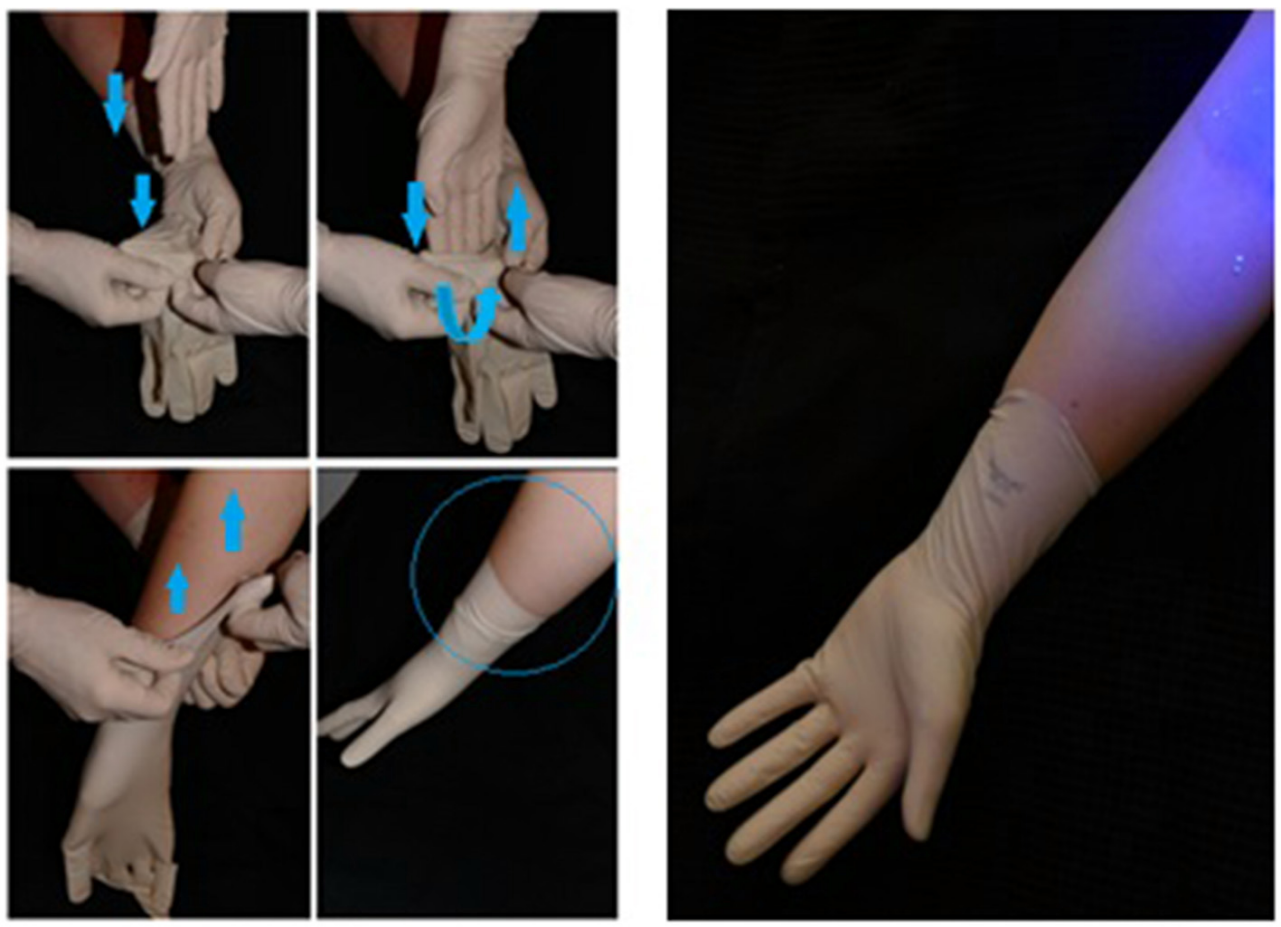
An example of how airborne contamination can occur. The aberrant air flow created with forceful entry of the hand into a glove can lead to airborne spread proximally.

From our experimental trials, 2 types of contamination were observed. As shown in Figure 4, evidence of direct contamination was seen where a sterile outer surface had come into direct contact with a Glitterbug™ powder-contaminated surface. As demonstrated by Figure 5, evidence of airborne spread was also seen as small dots of Glitterbug™ powder frequently appeared in trials along the length of the arm.

Table 2 shows the data for absolute contamination, i.e., those trials with any evidence of Glitterbug™ powder contamination. Method 2 (the unassisted method) had a lower contamination rate when compared to the other methods. Method 1 (the 3 pair method) had the most amount of contamination. This was shown to be statistically significant (*p* = 0.031).

**Table 2:** Absolute contamination pertains to any type of outer surface Glitterbug™ contamination present post-glove change.

**Absolute contamination**			
	**Method 1 (three pair)**	**Method 2 (unassisted)**	**Method 3 (nurse assist)**
Total number of trials:	64	65	81
Total number of contaminated trials:	59	49	64
Mean:	0.92	0.75	0.79
ANOVA: *p =* 0.031

When looking at the absolute contamination, a large proportion of the contaminations were due to airborne spread. That is, scant, small dots of Glitterbug™ powder were distantly seen up the arm up to a distance of 43 cm from the palmar crease.

Table 3 demonstrates the data pertaining to relative contamination, i.e., those trials with evidence of contamination caused only by direct contact. Method 3 (the nurse-assisted method) had the least number of contaminated trials. Method 1 (the 3 pair method) once again had the greatest number of contaminated trials.

**Table 3:** Relative contamination pertains to outer surface contamination of Glitterbug™ due to direct contact of a contaminated surface to a clean one (excluding trials with evidence of airborne spread).

**Relative contamination**			
	**Method 1 (three pair)**	**Method 2 (unassisted)**	**Method 3 (nurse assist)**
Total number of trials:	64	65	81
Total number of contaminated trials:	38	24	20
Mean:	0.59	0.37	0.25
ANOVA: *p* < 0.0001

Of the trials with evidence of direct contamination, there was no difference for handedness. There was no true pattern of contamination. However, the most soiled of the trials tended to be ones with smears of Glitterbug™ powder on the palmar aspect of the wrist, as seen in Figure 3. However, this was not statistically significant.

We found that there was no significant difference in the contamination rates between the trainees. However, we did observe that there was a trend in all 3 of our testing sessions (the pilot and 2 subsequent sessions). The first 6–8 trials for each participant had a lower proportion of contaminated trials, mostly via direct contamination. As the trials progressed, nearly every trial was contaminated, mostly with evidence of airborne spread.

## Discussion

In some surgical specialties where the development of an infection carries such significant consequences, it is almost routine practise that double gloves are worn to increase the barrier between surgeon and the surgical field. It also allows for, in the event of contamination, the ability to just remove the top glove only as long as the under glove has not been breached. In Orthopaedics, the number of arthroplasties being performed worldwide increase annually. Thus, there will be a subsequent increase in deep prosthetic joint infections (2, 4, 14). It has been well established that acute prosthetic joint infections are often due to exposure to pathogens at the time of index surgery, specifically by inoculation of the surgical site from skin flora (15). *Staphylococcus* aureus is the most common organism to cause deep prosthetic joint infections in Australia (40%) (16). There are numerous facets to the prevention of deep prosthetic joint infection (17, 18), and study has investigated just one aspect of infection prevention measures.

The use of sterile gloves is an area of little research, and there is no published data to date on top glove changing techniques. Most of the research centres around the risk of glove perforation and, certainly, the practise of double gloving is recommended by large, multicentre studies to minimise risk of perforation and complications from this (19). There is evidence which suggest that frequent glove changes after draping and during long cases helps to minimise contamination by removing gloves which may have been inadvertently contaminated or perforated (20). There is a potential of glove contamination during an operation, especially after draping, and frequent glove changing during operations has been recommended (21).

This present study confirms our initial hypothesis that Method 1, the three pair method, had the highest incidence of direct contamination due to the sterile surface of the under glove coming into contact with the contaminated outer surface of the remaining outer glove. Consideration has been given to potentially having assisted removal of top gloves in order to leave the under gloves of the surgeon uncontaminated. This could prove difficult in practise as it relies on having an assistant to help with glove removal, who will then potentially be contaminated themselves.

Method 2, the unassisted method, had less airborne spread than Method 3 (the nurse-assisted method) because there was less aberrant air flow created upon insertion of the hand within the glove. It has been shown that vigorous movements by personnel in the operating theatre leads to increased bacterial colonisation (22). Hence, it may be possible that decreasing the speed and force at which the hand is placed within the proffered glove would minimise such aberrant air flow and potential contamination.

There was less direct contamination with nurse-assisted gloving because the contaminated participant never touched the outside surface of the new outer glove. This result relies on the assumption that the scrub nurse who is assisting with the glove donning process is not contaminated.

One of the most interesting findings of this study was the surprisingly high proportion of contaminated trials, especially when assessing absolute contamination (trials with evidence of both airborne and direct contamination). Even Method 2, the unassisted method, which performed the best out of the 3 methods in terms of absolute contamination, still had 75% of trials contaminated. In reality, this result would translate to the surgeon having contaminated gloves in at least three quarters of all operations.

A potential limitation for this study could have been that the application of Glitterbug™ powder to the gloves was too “heavy handed.” In most situations, it would be unlikely that a surgeon would have their sterile outer surface so heavily soiled as the gloves were in this trial (Figure 1), thus potentially increasing local and airborne contamination. Only one study mentions the phenomenon of airborne Glitterbug™ particles impacting on contamination results (8). Upon literature review of airborne spread of small particles, it is likely that this is how *Staphylococcus* aureus and skin squamous cells behave (23, 24). The average pathogenic skin scale is around 14 μm, which is slightly larger than Glitterbug™ powder particles (1–5 μm). It is known that the smaller the particle, the more likely it is to remain airborne. This may therefore explain the higher than expected airborne contamination rate in this study (23). Despite this slight difference in size, Glitterbug™ powder has been widely used tool in the literature to simulate skin contamination (7–9).

Furthermore, this study was not performed in an operating theatre with ultraclean air filtration, which could have been the cause of increasing airborne contamination in later trials. With the turbulent airflow associated with movement of the participants around the trial room, without air filtration, the Glitterbug™ particles could have already been airborne and would already have contaminated the outer surface of the participants, irrespective of the trial method performed. Despite this, there still remained statistically significant differences of absolute contamination between the 3 methods of glove application.

What this study does show is that all three of the glove changing techniques are easy to perform. In addition, there does not appear to be a learning curve associated with them. Therefore, altering one's glove changing techniques is a small inconvenience for a large potential benefit of minimising surgical site contamination and preventing potential deep infection.

The most significant finding from the results is that Method 1, the three pair method, should be avoided in clinical practise. It is probably the most time efficient method to perform, but it was the method which performed the worst in terms of relative (59% of trials had evidence of direct contamination) and absolute contamination (92% of trials had evidence of direct or airborne contamination). However, recommendations as to which is the best method of glove changing is debateable. Methods 2 and 3 performed similarly. Method 2, the unassisted method, had more trials with direct contamination than Method 3, the nurse-assisted method (37% compared with 25%). However, when including the trials with airborne spread, Method 3 had slightly more contaminated trials than Method 2 (79% compared to 75%), most likely due to the force required to insert the hand into the glove held open by the nurse, which creates an eddy of airflow up the arm. This also relies on the assumption that the scrub nurse is not contaminated.

## Conclusion

Deep infections are a devastating complication of any surgery, and reducing surgical site contamination intra-operatively plays an important role in minimizing this risk. One potential strategy for minimizing intra-operative surgical site contamination is to modify the way surgeons change their gloves after real or potential contamination. The results of this blinded, prospective simulation demonstrate that Method 1, a technique involving wearing three pairs of gloves and then removing the outer layer, causes the most amount of outer surface contamination out of all of the glove changing techniques and cannot be recommended for use in surgery. The differences in contamination rates between Method 2 (wearing two pairs of gloves, removing the outer layer, and replacing them unassisted) and Method 3 (wearing two pairs of gloves, removing the outer layer, and replacing them with the assistance of a scrub nurse) are small. However, the results would suggest that Method 3 results in less direct contamination of the outer surface. This simulation study is the first to investigate glove changing techniques, and further *in vivo* research is recommended.

## Data Availability Statement

The raw data supporting the conclusions of this article will be made available by the authors, without undue reservation.

## Ethics Statement

The studies involving human participants were reviewed and approved by HREC Approval — Austin Health HREC — Trial 496. The patients/participants provided their written informed consent to participate in this study. Written informed consent was obtained from the individual(s) for the publication of any potentially identifiable images or data included in this article.

## Author Contributions

PB designed the study with assistance from EE, executed the ethics proposal, performed the experiment, collated the data, executed statistical analysis, and wrote the manuscript. EE proofread, revised the manuscript, and provided guidance on submission for publication. All authors contributed to the article and approved the submitted version.

## Conflict of Interest

The authors declare that the research was conducted in the absence of any commercial or financial relationships that could be construed as a potential conflict of interest.

## Publisher's Note

All claims expressed in this article are solely those of the authors and do not necessarily represent those of their affiliated organizations, or those of the publisher, the editors and the reviewers. Any product that may be evaluated in this article, or claim that may be made by its manufacturer, is not guaranteed or endorsed by the publisher.
